# Neoadjuvant Therapy in Pancreatic Cancer: An Emerging Strategy

**DOI:** 10.1155/2014/183852

**Published:** 2014-07-01

**Authors:** Alessandro Bittoni, Matteo Santoni, Andrea Lanese, Chiara Pellei, Kalliopi Andrikou, Cascinu Stefano

**Affiliations:** AOU Ospedali Riuniti, Polytechnic University of the Marche Region, Via Conca 71, 60126 Ancona, Italy

## Abstract

Pancreatic adenocarcinoma (PDAC) is the fourth leading cause of cancer deaths among men and women, being responsible for 6% of all cancer-related deaths. Surgical resection offers the only chance of cure, but only 15 to 20 percent of cases are potentially resectable at presentation. In recent years, increasing evidences support the use of neoadjuvant strategies in pancreatic cancer in patients with resectable pancreatic cancer as well as in patients with borderline resectable or locally advanced PDAC in order to allow early treatment of micrometastatic disease, tumour regression, and reduced risk of peritoneal tumour implantation during surgery. Furthermore, neoadjuvant treatment allows evaluation of tumour response and increases patient's compliance. However, most evidences in this setting come from retrospective analysis or small case series and in many studies chemotherapy or chemoradiation therapies used were suboptimal. Currently, prospective randomized trials using the most active chemotherapy regimens available are trying to define the real benefit of neoadjuvant strategies compared to conventional adjuvant strategies. In this review, the authors examined available data on neoadjuvant treatment in patients with resectable pancreatic cancer as well as in patients with borderline resectable or locally advanced PDAC and the future directions in this peculiar setting.

## 1. Introduction

Pancreatic ductal adenocarcinoma (PDAC) is an aggressive disease and still continues to have the worst prognosis of all gastrointestinal malignancies. Despite considerable advances in radiological techniques, it often presents as a locally advanced or metastatic disease in most patients and only about 10–20% of patients are considered candidate to surgery [1]. However, even in patients undergoing radical resection, the prognosis remains poor with a 5-year survival rate around 15–20% and a median overall survival (OS) in the order of 20–24 months due to the high rate of relapse [2]. In large series a 92% rate of disease relapse has been reported after PDAC resection without postoperative treatment. In particular, local recurrence occurs in about 40% of patients while distant metastases are observed as the only site of relapse in about 50% of cases, with liver as the primary site of distant relapse (36%) [3]. Margin involvement (R1) has been shown to be associated with poor prognosis in resected PDAC patients. Even in recent series a 15–35% rate of R1 resections has been reported, while macroscopically involved margins (R2) have been described in less than 1% of resections [4]. In spite of the improved outcomes observed after pancreaticoduodenectomy in the last years in high volume centers, the incidence of postoperative complications remains high (20–70%) while the mortality rate is between 1 and 4% [5].

Therefore surgery alone cannot be considered the optimal therapy for localized PDAC and complementary treatment; for example, chemotherapy and radiotherapy have been evaluated in the context of a multimodal approach.

Randomized studies have demonstrated the efficacy of adjuvant chemotherapy in patients with resected PDAC. Postoperative chemotherapy with gemcitabine provides a modest but significant survival benefit with a median OS of 23 months compared to about 20 months in patients undergoing resection alone and a 5-year survival rate of 21% versus 9% [3]. Similar results have been observed in clinical trials with adjuvant 5-fluorouracil [6]. On the contrary, data about adjuvant chemoradiotherapy in PDAC are more controversial. A few clinical trials have evaluated chemoradiation after surgery with conflicting results ranging from a significant improvement in OS [7] to a detrimental effect [6].

Overall, these results show how postoperative therapy can provide a significant but modest benefit to PDAC patients and highlight the need for more effective multimodal treatment strategies. Although the use of preoperative treatment, such as neoadjuvant chemotherapy or chemoradiotherapy, may represent an effective strategy for localized PDAC, limited evidences are currently available on this approach.

In this review we will overview available data on neoadjuvant treatment in patients with resectable pancreatic cancer as well as in patients with borderline resectable or locally advanced PDAC.

## 2. Neoadjuvant Therapy in Resectable Pancreatic Cancer

Neoadjuvant treatment has a strong rationale in pancreatic cancer and presents many theoretical advantages. Indeed, preoperative chemotherapy approach allows an early treatment of micrometastatic disease, responsible for relapse after curative surgery. Furthermore, a larger proportion of patients are able to complete the treatment in the preoperative compared to adjuvant setting. In fact, it has been shown by adjuvant trials that up to 25% of patients submitted to pancreatic resection do not receive the planned treatment due to postoperative complications, deterioration of performance status, comorbidities, or early recurrence [8]. Preoperative chemotherapy may also induce tumour regression, reducing the risk of R1 resection. Other potential advantages include better patients' tolerance to chemotherapy, a reduced risk of peritoneal tumour implantation during surgery, and the chance of an* in vivo* assessment of tumour chemosensitivity. Finally neoadjuvant treatment allows a better patient selection identifying those patients presenting with rapid progressive or disseminated disease at restaging who therefore have a very poor prognosis and for whom surgery is unlikely to provide any benefit.

During the last two decades, several studies have evaluated the role of neoadjuvant chemotherapy, radiotherapy, or combination of both in resectable pancreatic cancer.

Regarding chemotherapy, a phase II randomized trial allocated 50 patients with resectable PDAC to gemcitabine alone or to gemcitabine cisplatin. A 4% response rate was observed in patients treated with combination chemotherapy while no objective response was seen in patients treated with gemcitabine alone. Resection rate was higher in patients in the combination arm (70%) compared to patients in the gemcitabine arm (38%), while there were no differences in terms of surgical complications. Even overall survival (OS) was better in combination arm (15.6 months) compared with a median OS of 9.9 months in monotherapy arm [9]. In 28 resectable PDAC patients, receiving cisplatin and gemcitabine [10], a 89% rate of resectability was observed with 71% of R0 resections, not significantly different from the rate reported in the literature with surgery alone, and a median OS of 19.1 months for patients who underwent successful resection.

More data are available regarding neoadjuvant chemoradiotherapy. Indeed, most of the trials are single phase II studies. The administration of radiation therapy before surgery has some advantages compared to the postoperative setting and in particular it allows radiation to be delivered to well oxygenated cells before surgical devascularization. In a study performed at MD Anderson, 28 patients with resectable PDAC were treated with chemotherapy with 5-FU (300 mg/m^2^/day) concomitant to radiotherapy (50.4 Gy in 5.5 weeks). Gastrointestinal toxicity required hospitalization in 9 patients (32%) but no patients experienced a delay in surgery. A total of 23 patients without evidence of progressive disease underwent laparotomy and 17 patients (61%) a radical pancreatoduodenectomy. Perioperative complications occurred in three patients with one perioperative death [11]. Subsequently, the same group evaluated neoadjuvant chemoradiotherapy with gemcitabine concomitant to radiation therapy (30 Gy) on 86 patients with localized pancreatic cancer. The treatment included chemotherapy with gemcitabine (400 mg/m^2^ once a week for 7 weeks) concomitant to radiotherapy (30 Gy in 10 fractions in weeks 2 and 3). Pancreaticoduodenectomy was performed in 64 patients (75%) and 57 patients (66%) had R0 resection. Median OS for the whole patients population was 22.7 months while patients who underwent surgery had a median OS of 34 months. Main grade 3-4 toxicities observed included neutropenia, fatigue, nausea, and vomiting; there was no toxic death and all the patients concluded the planned treatment [12]. A phase II trial evaluated the combination of cisplatin and gemcitabine followed by gemcitabine-based chemoradiotherapy in 90 patients with resectable PDAC. Sixty-two (78%) of 79 patients who completed chemoradiation were taken to surgery and 52 (66%) of 79 underwent PD. Interestingly, margin involvement was described only in one patient, with a R1 resection rate (4%) significantly lower compared to data reported with surgery alone in the literature. The median OS of all 90 patients from the date of diagnosis was 17.4 months (95% CI, 14.5 to 20.3 months) while patients who completed chemoradiation and underwent surgery had a median OS of 31 months [13].

Also paclitaxel in combination with radiotherapy has been tested in patients with resectable PDAC. In a study on 35 patients, paclitaxel (weekly 3 h infusion of 60 mg/m^2^ for 3 consecutive weeks) concomitant to radiotherapy (30 Gy) was administered before surgery. Only 12 patients (34%) underwent R0 resection with a median OS of 19 months for the whole group and a high rate of distant failure (85%). On the whole, the results were less promising compared to what was observed with 5-FU based therapy [14]. Recently, results of a phase II trial evaluating gemcitabine and S-1 in this setting in an Asian population have been presented. The study included 36 resectable and borderline resectable PDAC patients treated with gemcitabine given at a dose of 1000 mg/m^2^ on days 1 and 8 of each cycle and S-1 administered orally at a dose of 40 mg/m^2^ twice daily for the first 14 consecutive days followed by a 7-day rest. The 2-year survival rate, primary endpoint of the study, was 45.7%. The R0 resection rate was quite high (87%) while the perioperative morbidity was 40%, in line with data from surgical series and then with no apparent increase in complication rates compared to surgery alone [15].

Overall, these studies showed that neoadjuvant chemoradiotherapy is a feasible approach and does not increase the risk of perioperative morbidity and mortality. Furthermore, patients who completed neoadjuvant chemoradiation and did not progress at restaging had a higher chance of achieving R0 resection and a higher overall survival when compared to historical data. Nevertheless, although chemoradiation has been shown to improve local control, it may not effectively decrease distant metastasis, as shown by the high rate of distant failure in these studies.

## 3. Neoadjuvant Therapy in Borderline Resectable and in Locally Advanced PDAC

As previously stated, only 10–20% of PDAC patients present with primarily resectable disease while locally advanced, nonmetastatic pancreatic cancer is seen in about 30% of patients [1] and median OS in this subgroup of patients is in the order of 9–13 months. Recently this group has been further subdivided by different authors into borderline resectable (BRPC) and locally advanced nonresectable (LAPC) pancreatic cancer.

### 3.1. Borderline Resectable Pancreatic Cancer: Definitions

Borderline resectable cancers have been* recently* defined as cancers with limited involvement of the mesenteric vessels, such that resection is technically possible, but which carry a high risk of margin-positive resection and consequently a higher risk of recurrence [22]. Therefore these tumours are distinct from both locally advanced and unresectable tumors, which are unlikely to be resectable to negative margins despite the use of induction therapy or complex reconstructive surgical techniques that from resectable tumors that are candidates for upfront pancreaticoduodenectomy. In this scenario, preoperative treatment may be specifically beneficial in borderline resectable PDAC, improving the fraction of patients undergoing radical resection.

Several anatomic definitions of borderline resectable pancreatic cancer have been given; however, the 3 most commonly cited definitions are those proposed by the MD Anderson group and the Americas Hepatopancreatobiliary Association (AHPBA)/Society for Surgery of the Alimentary Tract (SSAT)/Society of Surgical Oncology (SSO, modified by the NCCN) (Table 1).

The definition of borderline resectable tumours according to the NCCN guidelines includes the following characteristics: (i) no distant metastases; (ii) venous involvement of the superior mesenteric vein (SMV) or portal vein demonstrating tumour abutment with impingement and narrowing of the lumen, encasement of the SMV/portal vein, but without encasement of the nearby arteries, or short-segment venous occlusion resulting from either tumour thrombus or encasement, but with suitable vessel proximal and distal to the area of vessel involvement, allowing for safe resection and reconstruction; (iii) gastroduodenal artery encasement up to the hepatic artery with either short-segment encasement or direct abutment of the hepatic artery, without extension to the celiac axis; (iv) tumour abutment of the superior mesenteric artery (SMA) not to exceed >180° of the circumference of the vessel wall. By contrast, nonmetastatic pancreatic tumours are considered nonresectable if the following characteristics are fulfilled: (i) >180° SMA or celiac artery encasement; (ii) unreconstructable SMV/portal vein occlusion; (iii) aortic invasion or encasement; (iv) metastases to lymph nodes beyond the field of resection [23]. In addition to these radiological criteria, Katz et al. [24] introduced also patient-related factors in the concept of BRPC and proposed a classification in three subgroups: group A, defined by radiological criteria; group B, including patients with findings suggestive of metastases; and group C, including patients with comorbidities and marginal performance status.

### 3.2. Neoadjuvant Therapy in BRPC and LAPC

The optimal neoadjuvant therapy in patients with BRPC and LAPC remains a matter of debate due to the lack of randomized studies. In both categories local tumour reduction and systemic control represent primary goals of treatment and then common strategies may be applied in both entities. Moreover, in many studies these two entities have been evaluated together.

Chemoradiotherapy is a common experimental approach therapy in BRPC. Massucco et al. [25] evaluated 28 patients with BRPC and nonresectable pancreatic cancer who received gemcitabine-based chemoradiotherapy. While only 1 out of 8 patients with initially unresectable disease underwent resection, 7 out of 18 (39%) of BRPC were resected. Chemoradiotherapy did not increase perioperative morbidity and mortality. Median overall survival for the whole group was 15 months. In both groups, a disease-free survival beyond 24 months was observed in patients resected with negative margins. A different strategy was evaluated by Patel et al. [26] on seventeen patients with BRPC. The patients were treated with three cycles of induction chemotherapy with gemcitabine, docetaxel, and capecitabine followed by 5-FU based chemoradiotherapy with IMRT. Eleven patients (64.7%) out of 17 underwent resection and eight patients (47%) achieved an R0 resection. The median progression-free survival and OS were 10.48 months and 15.64 months, respectively. Stokes et al. [27] also prospectively examined 40 borderline resectable pancreatic cancer patients treated with combined capecitabine-based chemoradiation. Of these, 34 (85%) completed neoadjuvant treatment and were restaged. A total of 16 patients (46%) proceeded to surgery, with 88% with an R0 resection and median overall survival of 23 months. Kim et al. evaluated a chemoradiotherapy regimen including gemcitabine and oxaliplatin on 68 BRPC and LAPC patients: after the treatment, completed by 90% of patients, forty-three patients underwent resection (63%), and R0 resection was achieved in 36 of those 43 patients (84%). The median overall survival was 18.2 months for all patients and 27.1 months for those who underwent resection [28].

The benefit of neoadjuvant therapies in BRPC was retrospectively reviewed by Katz et al. [24] at MDACC. Between 1999 and 2006, 160 (7%) of 2454 pancreatic cancer patients were classified as having borderline resectable disease and were scheduled to receive 2–4 months of neoadjuvant chemotherapy followed by radiation in combination with either 5-fluorouracil (5-FU), gemcitabine, capecitabine, or paclitaxel. Restaging CT scan was repeated every 2 months during the treatment and 4 to 6 weeks after completion to determine resectability. Patients who experienced disease progression or had deterioration of performance status during this period of treatment were excluded from surgery. One hundred twenty-five (78%) patients completed the restaging, 79 (63% of 125) patients proceeded to surgery, and 66 (53% of 125) patients received pancreaticoduodenectomy. For the whole patients population (160 patients) with borderline resectable disease, the median OS was 18 months and the 5-year survival was 18%. Importantly, the 66 patients who completed the whole therapy including surgery had a significantly better clinical outcome (median OS of 40 months and a 5-year survival rate of 36%) compared to a median survival of 13 months in the remaining 94 unresected patients. Patients with greater pathologic response or drop in serum CA19-9 levels during neoadjuvant therapy had better OS. However, 59% of the resected patients had a recurrence, mainly occurring in distant organs such as lung, liver, or bone (45%); 9% had recurrence in the pancreatic bed; and 11% had recurrence in the peritoneum or regional lymph nodes. These results confirm a positive effect of neoadjuvant treatment in terms of resection rates and long-term survival in patients with BRPC. However, the high rates of disease relapse claim for more effective treatments.

Data about the efficacy of chemotherapy in LAPC mainly come from subgroup analysis of studies in advanced pancreatic cancer. Most of the studies investigating the efficacy of gemcitabine-based chemotherapy in advanced pancreatic cancer included a percentage of LAPC patients. Gemcitabine-based combinations have proved to induce higher response rate (about 26%) compared to single agent gemcitabine (4–15%) and response rates were similar to those observed in metastatic disease [29, 30]. A phase II trial, the NeoGemOx trial, evaluated gemcitabine and oxaliplatin combination in 33 LAPC patients. After treatment, 39% of patients underwent curative resection, with a 69% of R0 resections. Median OS of patients who underwent tumor resection was 22 months compared with 12 months for those without resection (*P* = 0.046). The study confirmed that the combination of gemcitabine and oxaliplatin is active in LAPC patients and induces tumour regression in a significant proportion of patients [31]. Also the combination of gemcitabine and capecitabine has been assessed in this subset of patients. In a study by Lee et al., forty-three patients (18 with BRPC and 25 with unresectable disease) were treated with fixed-dose rate gemcitabine and capecitabine. Surgery was performed in 17 patients (39.5%); pathologic radical resection (R0) was achieved in 82.3% among the 17 resected patients. Median OS was 23.1 months in patients undergoing surgery [32]. An Italian study evaluated an upfront intensive chemotherapy combination followed by chemoradiotherapy in the treatment of LAPC. In particular, patients received PEFG/PEXG (cisplatin, epirubicin, 5-fluorouracil/capecitabine, and gemcitabine) or PDXG (docetaxel substituting epirubicin) regimen for 6 months followed by radiotherapy (50–60 Gy) with concurrent gemcitabine or fluoropyrimidines. A high response rate was observed (47%) while stable disease was reported in 42% of patients. Thirteen patients of ninety-one included in the analysis (14%) were radically resected yielding one pathologic complete remission [33]. The use of CRT alone in LAPC has been evaluated in different studies. One randomized study demonstrated the superiority of 5-fluorouracil (5-FU) based CRT compared with best supportive care. Most studies used 5-FU or gemcitabine as reference chemotherapy in combination with radiation doses of 50–60 Gy. A recent meta-analysis suggested that the combination of radiation with gemcitabine might be more effective than the combination with 5-FU [34].

A recent systematic review has evaluated 111 trials including 4394 pancreatic cancer patients [35]. Neoadjuvant therapy included chemotherapy in 96% and radiation therapy in 94% of studies. For nonresectable patients, the estimated overall response rate was 35%. Among patients with initially nonresectable tumours, surgical exploration was performed in 47% of cases. The overall resection rate after neoadjuvant therapy was 33%, and 79% of resections were R0 resections. Median OS of the 33% resected patients was 20.5 months while unresectable patients, who were not resected, had a median OS of 10.2 months. This analysis suggests that neoadjuvant treatment may be able to induce conversion to resectability in about one-third of LAPC patients and, importantly, tumour resection is associated with significantly prolonged overall survival, comparable to what was observed in primarily resectable pancreatic cancer patients.

### 3.3. New Treatment Strategies

Recently FOLFIRINOX, a three-drug combination regimen including oxaliplatin, irinotecan, leucovorin, and 5-FU, was shown to be superior to gemcitabine in patients with metastatic pancreatic cancer, with a median OS of 11.1 months in the FOLFIRINOX group versus 6.8 months in the gemcitabine group (*P* < 0.001) and a median progression-free survival of 6.4 months versus 3.3 months (*P* < 0.001), although FOLFIRINOX toxicity was higher [36]. Interestingly, FOLFIRINOX demonstrated also a higher response rate compared to gemcitabine (32% versus 9%, *P* < 0.001). Notably, the phase III study by Conroy et al. included only patients with metastatic disease and then results cannot be translated to LAPC patients. However, preliminary data on the efficacy of FOLFIRINOX regimen in LAPC patients are available from some retrospective analysis or small case series (Table 2).

A retrospective analysis conducted by Hosein et al. [16] on 18 patients with borderline resectable and unresectable pancreatic cancer showed a 28% rate of R0 resection after chemotherapy. Among the 11 patients who remained unresectable after FOLFIRINOX, 3 went on to have R0 resections after combined chemoradiotherapy, for an overall R0 resection rate of 44%. An analysis reported by Gunturu et al. [17] included 16 patients with LAPC treated with FOLFIRINOX. A response rate of 50% was shown in LAPC patients; interestingly, the authors modified the FOLFIRINOX regimen with dose reductions in 29 out of 35 patients starting from the first cycle. This dose attenuation of irinotecan and bolus fluorouracil was shown to improve tolerability without compromising efficacy. Similar results were reported by Peddi et al. [18] in a multi-institutional registry study. Among 23 patients with BRPC and LAPC a 34% response rate was observed, with an 84% disease control rate, in spite of dose reductions. In particular deletion of 5-FU and dose reduction of irinotecan were the most common modifications applied.

More recently, results of a retrospective analysis conducted on 43 patients with BRPC and LAPC treated with a modified FOLFIRINOX regime have been presented [19]. The regimen used in the study had no bolus 5-FU and a lower dose of irinotecan compared to the regimen evaluated by Conroy. Overall resection rate was 53.8% including 45% of patients with initially unresectable disease and R0 resection was achieved in 85.7% of the resected patients. The median PFS of resected patients in this analysis reached 18.4 months. All patients received prophylactic pegfilgrastim and rate of G3/4 hematological toxicities was remarkably low with no episode of febrile neutropenia or G3/4 thrombocytopenia. A similar chemotherapy combination, the FOLFOXIRI regimen, has been evaluated in an Italian phase II study in 32 patients with unresectable or borderline resectable PDAC patients. FOLFOXIRI consisted of a lower dose of irinotecan (150 mg/m^2^) and of infusional 5-fluorouracil (2800 mg/m^2^ as a 48-hour continuous infusion on days 1 to 3) compared to FOLFIRINOX with no bolus 5-fluorouracil, while folinic acid and oxaliplatin (85 mg/m^2^) remained unchanged. The FOLFOXIRI regimen was active, with a 37% objective response rate, and allowed radical resection in 41% of patients with a median OS for the patients enrolled of 24.2 months [20].

Nab-paclitaxel, an albumin bound formulation of paclitaxel particles, has recently been shown to be effective in the treatment of advanced pancreatic cancer. The MPACT trial, comparing nab-paclitaxel and gemcitabine versus gemcitabine alone, found a significant increase in median OS (8.5 months versus 6.7, *P* < 0.001) in PFS (median PFS 5.5 months versus 3.7 months, *P* < 0.001) and in overall response rate (23% versus 7%, *P* < 0.001) for the combination [37]. This trial included only patients with metastatic disease; therefore data about the efficacy of nab-paclitaxel in patients with locally advanced disease are lacking. However, considering the hypothesis that the antitumour effect of nab-paclitaxel is mediated by depletion of peritumoural stroma and improved transport of chemotherapeutic agents to the tumour, the use of this drug in localized disease in the neoadjuvant setting appears to be promising. In 2013, preliminary results of a pilot study of sequential neoadjuvant chemotherapy with nab-paclitaxel plus gemcitabine followed by FOLFIRINOX in locally advanced pancreatic cancer were presented [21]. Eight LAPC patients received 2 cycles of nab-paclitaxel and gemcitabine followed by 2 cycles of FOLFIRINOX. All patients received the planned 4 cycles of neoadjuvant chemotherapy without dose reductions and there were no treatment-related deaths and none of the patients stopped treatment due to toxicity. Among the 8 evaluable patients, 5 partial responses (63%) and 3 stable disease (37%) were observed, resulting in a disease control rate of 100%. After sequential chemotherapy 3  patients (37%) underwent radical surgical resection. Notably, all resected tumours showed signs of tumour regression with one patient showing a complete pathological response.

Although data about the efficacy of new treatment regimens in neoadjuvant setting are promising, prospective studies are required to confirm the efficacy and the tolerability of FOLFIRINOX and nab-paclitaxel in BRPC and LAPC.

## 4. Ongoing Trial in Neoadjuvant Setting for PDAC

Among the 1607 studies running in patients with pancreatic cancer, 91 studies are focused on neoadjuvant chemotherapy. A selection of these trials is summarized in Table 3. Several studies are assessing the efficacy of gemcitabine alone or in combination with other drugs in this setting. Among them, a multicenter prospective randomized phase II/III study of neoadjuvant chemoradiation with gemcitabine is ongoing in patients with borderline resectable pancreatic cancer. This study is designed in 2 arms, one with upfront surgery and the other with neoadjuvant chemoradiation therapy (NCT01458717).

Another randomized phase III study (NEOPAC study, NCT01314027) is comparing adjuvant gemcitabine and neoadjuvant gemcitabine/oxaliplatin plus adjuvant gemcitabine in resectable pancreatic cancer. On the other hand, the NEOPA study is a phase III trial of chemoradiation with weekly gemcitabine 300 mg/m^2^ for 6 weeks combined with external beam radiotherapy (EBRT) followed by surgery and adjuvant gemcitabine (1000 mg/m^2^ 6 cycles at days 1, 8, and 15 of each 28-day cycle) versus upfront surgery followed by adjuvant gemcitabine (NCT01900327).

Moreover, a phase I study is evaluating the association of neoadjuvant hypofractionated chemoradiation with gemcitabine plus radiosurgical boost for patients with borderline resectable and locally advanced unresectable pancreatic cancer (RT-054, NCT01739439).

Furthermore, a phase I study of preoperative gemcitabine plus CD40 agonist antibody CP-870,893 followed by addition of CP-870,893 to standard of care adjuvant chemoradiation is recruiting patients with newly diagnosed resectable PDAC (NCT01456585).

Gemcitabine is also under evaluation in a phase II study in combination with capecitabine followed by SBRT for potentially resectable, locally advanced PDAC (NCT01360593) and in a phase II study in combination with erlotinib before and after pancreatectomy for patients with operable PDAC (NCT00733746).

Abraxane represents one of the most promising agents for patients with pancreatic cancer. With regard to its use in the neoadjuvant setting, the NEONAX randomized phase II trial is in course to compare neoadjuvant plus adjuvant or only adjuvant nab-paclitaxel plus gemcitabine for resectable pancreatic cancer (NCT02047513). This combination is under evaluation also in an open-label phase 1/2 study that will combine gemcitabine and nab-paclitaxel with an oral hedgehog inhibitor LDE225 in patients with borderline resectable PDAC (NCT01431794).

Presently, six studies are assessing the potential of FOLFIRINOX as neoadjuvant therapy for pancreatic cancer. Among them, a phase II trial has been designed with FOLFIRINOX (5-fluorouracil, irinotecan, oxaliplatin, and gemcitabine) for six cycles prior to combined modality treatment with gemcitabine during and following IMRT (NCT01661088).

In addition, two phase II trials are ongoing with FOLFIRINOX and chemoradiation followed by definitive surgery (NCT01677988) or definitive surgery and postoperative gemcitabine for patients with borderline resectable pancreatic adenocarcinoma (NCT01821612).

Moreover, two phase II studies are assessing the efficacy and safety of pre- and postsurgery FOLFIRINOX in patients with localized pancreatic cancer (NCT01660711, NCT02047474).

Finally, a phase I study of stereotactic body radiation therapy (SBRT) and neoadjuvant FOLFIRINOX is ongoing in resectable pancreatic cancer (NCT01446458).

Several trials are in course to evaluate the use of capecitabine as neoadjuvant therapy for pancreatic cancer patients. Among them, a phase II trial has been opened to evaluate the efficacy and safety of the combination of capecitabine, oxaliplatin, and irinotecan (CAPOXIRI) in patients with resectable, borderline resectable, and locally advanced PDAC (NCT01760252). In addition, a phase II study is investigating the role of neoadjuvant proton beam radiation therapy and concomitant capecitabine in marginally resectable carcinoma of the pancreas (NCT00763516). Another phase II study of neoadjuvant accelerated short course radiation therapy with proton beam capecitabine and hydroxychloroquine (NCT01494155) and a randomized phase II/III trial, testing the efficacy and safety of peri- or postoperative chemotherapy with capecitabine, cisplatin, epirubicin, and gemcitabine in resectable PDAC (NCT01150630), are enrolling patients. Finally, a phase II/III nonrandomized study has been designed to assess the safety and benefit of 6 cycles of chemotherapy treatment consisting of gemcitabine, capecitabine, and docetaxel (also called “GTX”). In group I, patients with only venous involvement receive 6 cycles of gemcitabine, capecitabine, and docetaxel (GTX) and then surgery. In group II, patients with arterial involvement and/or venous involvement receive 6 cycles of GTX, then GX/RT and then surgery (NCT01065870). Another phase II trial is ongoing to evaluate the efficacy and safety of SBRT in combination with GTX in patients with borderline resectable PDAC (NCT01754623).

Even immunotherapeutic approaches are considered as neoadjuvant therapies. A phase II study of neoadjuvant chemotherapy (gemcitabine and fluorouracil) with and without immunotherapy to CA125 (Oregovomab) followed by hypofractionated stereotactic radiotherapy and concurrent HIV protease inhibitor Nelfinavir is in course in patients with locally advanced pancreatic cancer (NCT01959672).

Finally, a phase II study is evaluating the combination of fluorouracil prodrug Tegafur, leucovorin, and concomitant RT with or without cetuximab in patients with locally advanced pancreatic cancer (PERU, NCT01050426). In addition, the NEOPANC trial has been designed as a prospective, one armed single center study to investigate neoadjuvant short course intensity-modulated radiation therapy (IMRT) in combination with surgery and intraoperative radiation therapy (IORT) followed by adjuvant chemotherapy in patients with primarily resectable pancreatic cancer (NCT01372735).

## 5. Conclusions 

Increasing evidences support the use of neoadjuvant strategies in pancreatic cancer. Nevertheless, the role of neoadjuvant therapy in patients with resectable pancreatic cancer has not yet been defined. Most of the available evidences derive from retrospective analysis or small case series, and in many studies chemotherapy or chemoradiation therapies used were suboptimal. Prospective and controlled randomized trials using the most active chemotherapy regimens currently available are warranted to assess the benefit of neoadjuvant strategies compared to conventional adjuvant strategies in this setting. Presently, the use of neoadjuvant therapies in patients with resectable pancreatic cancer could be considered in the context of a multidisciplinary approach, within clinical trials or for patients with high risk of early relapse. In particular, it has been demonstrated that high CA 19.9 serum levels (CA 19.9 > 200 U/mL), long duration of preoperative symptoms (>40 days), and pathological grading (G3-G4) are associated with high risk of early relapse and then may be used to identify patients who may benefit from preoperative chemotherapy [38].

In patients with borderline resectable or nonresectable pancreatic cancer, neoadjuvant therapy may achieve downsizing of the tumour, increasing the probability of R0 resections, or may convert the tumour to become resectable. Moreover, in patients with BRPC neoadjuvant chemotherapy may also be able to identify a subgroup of patients with early progression and so being unlikely to benefit from surgery. Currently available data do not allow defining an optimal regimen in this setting. Combination chemotherapy appears to achieve higher response rates than single-agent chemotherapy, while there are no sufficient evidences to show that chemoradiotherapy is superior to chemotherapy alone. Further options are arising, with the development of new and more effective chemotherapeutic regimens, namely, FOLFIRINOX and nab-paclitaxel. However, the efficacy of these treatments in neoadjuvant setting needs to be verified in prospective clinical trials.

## Figures and Tables

**Table 1:** Comparison of definitions of borderline resectable pancreatic cancer.

	AHBPA/SSAT/SSO	MD Anderson	NCCN 2012
SMV-PV	Abutment, encasement, or occlusion	Occlusion	Abutment with impingement and narrowing

SMA	Abutment	Abutment	Abutment

CHA	Abutment or short-segment encasement	Abutment or short-segment encasement	Abutment or short-segment encasement

Celiac trunk	No abutment or encasement	Abutment	No abutment or encasement

SMV-PV: superior mesenteric vein-portal vein; SMA: superior mesenteric artery.

CHA: common hepatic artery.

**Table 2:** FOLFIRINOX regimen in patients with borderline resectable or locally advanced unresectable pancreatic cancer.

Author	Study design	*n* pts	ORR	Resection rate	R0 resections	1-year PFS
Hosein et al. [16]	Retrospective	18	—	39%	28%	83%
Gunturu et al. [17]	Retrospective	16	50%	—	—	—
Peddi et al. [18]	Registry study	23	34%	—	—	75%
Blazer et al. [19]	Retrospective	43	—	54%	42%	—
Vasile et al. [20]	Phase II	32	37%	41%	—	—
Kunzmann et al. [21]	Phase II∗	8	63%	37%	—	—

*Sequential regimen including FOLFIRINOX and nab-paclitaxel plus gemcitabine.

**Table 3:** Ongoing clinical trials in neoadjuvant setting in PDAC.

Treatment	Setting	Trial identification number	Phase	Design
Gemcitabine	BRPC	NCT01458717	II/III	Upfront surgery versus neoadjuvant gemcitabine-based chemoradiation therapy

Gemcitabine/Oxaliplatin	Resectable PC	NCT01314027	III	Adjuvant gemcitabine versus neoadjuvant gemcitabine/oxaliplatin plus adjuvant gemcitabine

Gemcitabine + RT	Resectable PC	NCT01900327	II	Chemoradiation with gemcitabine + RT followed by surgery and adjuvant gemcitabine versus upfront surgery plus adjuvant gemcitabine

Gemcitabine + RT	BRPC and LAPC	NCT01739439	I	Hypofractionated chemoradiation with gemcitabine plus radiosurgical boost

Gemcitabine + CP-870,893 + RT	Resectable PC	NCT01456585	I	Gemcitabine plus CD40 agonist antibody CP-870,893 followed by addition of CP-870,893 versus adjuvant chemoradiation

Gemcitabine + capecitabine	Resectable PC	NCT01360593	II	Gemcitabine + capecitabine + RT

Gemcitabine + erlotinib	Resectable PC	NCT00733746	II	Gemcitabine + erlotinib before and after surgery

Nab-paclitaxel + gemcitabine	Resectable PC	NCT02047513	III	Neoadjuvant plus adjuvant or only adjuvant nab-paclitaxel plus gemcitabine for resectable pancreatic cancer

Nab-paclitaxel + gemcitabine + LDE225	BRPC	NCT01431794	II	Gemcitabine and nab-paclitaxel with LDE225 (oral hedgehog inhibitor)

FOLFIRINOX	Resectable PC	NCT01661088	II	FOLFIRINOX followed by combined modality treatment with gemcitabine during and following RT

FOLFIRINOX	Resectable PC	NCT01677988	II	FOLFIRINOX and chemoradiation followed by surgery

FOLFIRINOX	BRPC	NCT01821612	II	FOLFIRINOX and chemoradiation followed by surgery and adjuvant gemcitabine

FOLFIRINOX	Resectable PC	NCT01446458	I	RT and neoadjuvant FOLFIRINOX

CAPOXIRI	Resectable PC, BRPC, and LAPC	NCT00087022	II	Neoadjuvant capecitabine, oxaliplatin, and irinotecan (CAPOXIRI)

Capecitabine + RT	BRPC	NCT00763516	II	Neoadjuvant proton beam radiation therapy and concomitant capecitabine

capecitabine and hydroxychloroquine + RT	Resectable PC	NCT01494155	II	Neoadjuvant accelerated short course RT with proton beam capecitabine and hydroxychloroquine

capecitabine, cisplatin, epirubicin, and gemcitabine	Resectable PC	NCT01150630	II/III	Peri- or postoperative chemotherapy with capecitabine, cisplatin, epirubicin, and gemcitabine

GTX + RT	BRPC	NCT01065870	II/III	Gemcitabine, capecitabine, and docetaxel (GTX) versus GTX + RT

GTX + RT	BRPC	NCT01754623	II	RT in combination with GTX

Gem, 5-FU Oregovomab, Nelfinavir + RT	LAPC	NCT01959672	II	Gemcitabine and 5-FU with and without immunotherapy (Oregovomab) followed by RT and Nelfinavir

Tegafur, cetuximab + RT	LAPC	NCT01050426	II	Tegafur, leucovorin, and concomitant RT with or without cetuximab

IMRT + IORT	Resectable PC	NCT01372735	II	IMRT in combination with surgery and intraoperative radiation therapy (IORT) followed by adjuvant chemotherapy

## References

[1] Hidalgo M (2010). Pancreatic cancer. *The New England Journal of Medicine*.

[2] Ueno H, Kosuge T, Matsuyama Y (2009). A randomised phase III trial comparing gemcitabine with surgery-only in patients with resected pancreatic cancer: Japanese Study Group of Adjuvant Therapy for Pancreatic Cancer. *British Journal of Cancer*.

[3] Oettle H, Post S, Neuhaus P (2007). Adjuvant chemotherapy with gemcitabine vs observation in patients undergoing curative-intent resection of pancreatic cancer: a randomized controlled trial. *Journal of the American Medical Association*.

[4] Chang DK, Johns AL, Merrett ND (2009). Margin clearence and outcome in resected pancreatic cancer. *Journal of Clinical Oncology*.

[5] Sukharamwala P, Thoens J, Szuchmacher M (2012). Advanced age is a risk factor for post-operative complications and mortality after a pancreaticoduodenectomy: a meta-analysis and systematic review. *HPB*.

[6] Neoptolemos JP, Stocken DD, Friess H (2004). A Randomized Trial of Chemoradiotherapy and Chemotherapy after Resection of Pancreatic Cancer. *The New England Journal of Medicine*.

[7] Kalser MH, Ellenberg SS (1985). Pancreatic cancer. Adjuvant combined radiation and chemotherapy following curative resection. *Archives of Surgery*.

[8] Klinkenbijl JH, Jeekel J, Sahmoud T (1999). Adjuvant radiotherapy and 5-fluorouracil after curative resection of cancer of the pancreas and periampullary region: phase III trial of the EORTC Gastrointestinal Tract Cancer Cooperative Group. *Annals of Surgery*.

[9] Palmer DH, Stocken DD, Hewitt H (2007). A randomized phase 2 trial of neoadjuvant chemotherapy in resectable pancreatic cancer: gemcitabine alone versus gemcitabine combined with cisplatin. *Annals of Surgical Oncology*.

[10] Heinrich S, Pestalozzi BC, Schäfer M (2008). Prospective phase II trial of neoadjuvant chemotherapy with gemcitabine and cisplatin for resectable adenocarcinoma of the pancreatic head. *Journal of Clinical Oncology*.

[11] Evans DB, Rich TA, Byrd DR (1992). Preoperative chemoradiation and pancreaticoduodenectomy for adenocarcinoma of the pancreas. *Archives of Surgery*.

[12] Evans DB, Varadhachary GR, Crane CH (2008). Preoperative gemcitabine-based chemoradiation for patients with resectable adenocarcinoma of the pancreatic head. *Journal of Clinical Oncology*.

[13] Varadhachary GR, Wolff RA, Crane CH (2008). Preoperative gemcitabine and cisplatin followed by gemcitabine-based chemoradiation for resectable adenocarcinoma of the pancreatic head. *Journal of Clinical Oncology*.

[14] Pisters PWT, Wolff RA, Janjan NA (2002). Preoperative paclitaxel and concurrent rapid-fractionation radiation for resectable pancreatic adenocarcinoma: Toxicities, histologic response rates, and event-free outcome. *Journal of Clinical Oncology*.

[15] Mizuma M, Motoi F, Ishida K (2014). Neoadjuvant chemotherapy with gemcitabine and S-1 for resectable and borderline pancreatic ductal adenocarcinoma: a prospective, multi-institutional, phase II trial. *Journal of Clinical Oncology*.

[16] Hosein PJ, Macintyre J, Kawamura C (2012). A retrospective study of neoadjuvant FOLFIRINOX in unresectable or borderline-resectable locally advanced pancreatic adenocarcinoma. *BMC Cancer*.

[17] Gunturu KS, Yao X, Cong X (2013). FOLFIRINOX for locally advanced and metastatic pancreatic cancer: single institution retrospective review of efficacy and toxicity. *Medical Oncology*.

[18] Peddi PF, Lubner S, McWilliams R (2012). Multi-institutional experience with FOLFIRINOX in pancreatic adenocarcinoma. *Journal of the Pancreas*.

[19] Blazer MA, Wu C, Goldberg R (2014). Tolerability and efficacy of modified FOLFIRINOX (mFOLFIRINOX) in patients with borderline-resectable pancreatic cancer (BRPC) and locally advanced unresectable pancreatic cancer (LAURPC). *Journal of Clinical Oncology*.

[20] Vasile E, de Lio N, Cappelli C (2013). Phase II study of neoadjuvant chemotherapy with modified FOLFOXIRI in borderline resectable or unresectable stage III pancreatic cancer. *Journal of Clinical Oncology*.

[21] Kunzmann V, Hartlapp I, Scheurlen M (2013). Sequential neoadjuvant chemotherpy with nab-paclitaxel plus gemcitabine and FOLFIRINOX in locally advanced pancreatic cancer (LAPC): a PILOT study. *Journal of Clinical Oncology*.

[22] Katz MHG, Marsh R, Herman JM (2013). Borderline resectable pancreatic cancer: need for standardization and methods for optimal clinical trial design. *Annals of Surgical Oncology*.

[23] NCCN guidelines Pancreatic adenocarcinoma.

[24] Katz MHG, Pisters PWT, Evans DB (2008). Borderline resectable pancreatic cancer: the importance of this emerging stage of disease. *Journal of the American College of Surgeons*.

[25] Massucco P, Capussotti L, Magnino A (2006). Pancreatic resections after chemoradiotherapy for locally advanced ductal adenocarcinoma: analysis of perioperative outcome and survival. *Annals of Surgical Oncology*.

[26] Patel M, Hoffe S, Malafa M (2011). Neoadjuvant GTX Chemotherapy and IMRT-based chemoradiation for borderline resectable pancreatic cancer. *Journal of Surgical Oncology*.

[27] Stokes JB, Nolan NJ, Stelow EB (2011). Preoperative capecitabine and concurrent radiation for borderline resectable pancreatic cancer. *Annals of Surgical Oncology*.

[28] Kim EJ, Ben-Josef E, Herman JM (2013). A multi-institutional phase 2 study of neoadjuvant gemcitabine and oxaliplatin with radiation therapy in patients with pancreatic cancer. *Cancer*.

[29] Louvet C, Labianca R, Hammel P (2005). Gemcitabine in combination with oxaliplatin compared with gemcitabine alone in locally advanced or metastatic pancreatic cancer: results of a GERCOR and GISCAD phase III trial. *Journal of Clinical Oncology*.

[30] Lima CMR, Green MR, Rotche R (2004). Irinotecan plus gemcitabine results in no survival advantage compared with gemcitabine monotherapy in patients with locally advanced or metastatic pancreatic cancer despite increased tumor response rate. *Journal of Clinical Oncology*.

[31] Sahora K, Kuehrer I, Eisenhut A (2011). NeoGemOx: gemcitabine and oxaliplatin as neoadjuvant treatment for locally advanced, nonmetastasized pancreatic cancer. *Surgery*.

[32] Lee JL, Kim SC, Kim JH (2012). Prospective efficacy and safety study of neoadjuvant gemcitabine with capecitabine combination chemotherapy for borderline-resectable or unresectable locally advanced pancreatic adenocarcinoma. *Surgery*.

[33] Reni M, Cereda S, Balzano G (2009). Outcome of upfront combination chemotherapy followed by chemoradiation for locally advanced pancreatic adenocarcinoma. *Cancer Chemotherapy and Pharmacology*.

[34] Zhu CP, Shi J, Chen YX, Xie W, Lin Y (2011). Gemcitabine in the chemoradiotherapy for locally advanced pancreatic cancer: a meta-analysis. *Radiotherapy and Oncology*.

[35] Gillen S, Schuster T, Büschenfelde CMZ, Friess H, Kleeff J (2010). Preoperative/neoadjuvant therapy in pancreatic cancer: A systematic review and meta-analysis of response and resection percentages. *PLoS Medicine*.

[36] Conroy T, Desseigne F, Ychou M (2011). FOLFIRINOX versus gemcitabine for metastatic pancreatic cancer. *The New England Journal of Medicine*.

[37] von Hoff DD, Ervin T, Arena FP (2013). Increased survival in pancreatic cancer with nab-paclitaxel plus gemcitabine. *New England Journal of Medicine*.

[38] Barugola G, Partelli S, Marcucci S (2009). Resectable pancreatic cancer: who really benefits from resection?. *Annals of Surgical Oncology*.

